# Plasma radio-metabolite analysis of PET tracers for dynamic PET imaging: TLC and autoradiography

**DOI:** 10.1186/s13550-020-00705-2

**Published:** 2020-11-23

**Authors:** Fiona Li, Justin W. Hicks, Lihai Yu, Lise Desjardin, Laura Morrison, Jennifer Hadway, Ting-Yim Lee

**Affiliations:** 1 Department of Medical Biophysics, The University of Western University, 1151 Richmond Street North, London, ON N6A 3K7 Canada; 2 Lawson Health Research Institute, Grosvenor Campus, 268 Grosvenor Street, London, ON N6A 4V2 Canada; 3 Robarts Research Institute, London, ON Canada

**Keywords:** [^18^F]FEPPA, [^18^F]FAZA, Radio-metabolite correction, Thin layer chromatography (TLC), Dynamic PET, Autoradiography

## Abstract

**Background:**

In molecular imaging with dynamic PET, the binding and dissociation of a targeted tracer is characterized by kinetics modeling which requires the arterial concentration of the tracer to be measured accurately. Once in the body the radiolabeled parent tracer may be subjected to hydrolysis, demethylation/dealkylation and other biochemical processes, resulting in the production and accumulation of different metabolites in blood which can be labeled with the same PET radionuclide as the parent. Since these radio-metabolites cannot be distinguished by PET scanning from the parent tracer, their contribution to the arterial concentration curve has to be removed for the accurate estimation of kinetic parameters from kinetic analysis of dynamic PET. High-performance liquid chromatography has been used to separate and measure radio-metabolites in blood plasma; however, the method is labor intensive and remains a challenge to implement for each individual patient. The purpose of this study is to develop an alternate technique based on thin layer chromatography (TLC) and a sensitive commercial autoradiography system (Beaver, Ai4R, Nantes, France) to measure radio-metabolites in blood plasma of two targeted tracers—[^18^F]FAZA and [^18^F]FEPPA, for imaging hypoxia and inflammation, respectively.

**Results:**

Radioactivity as low as 17 Bq in 2 µL of pig’s plasma can be detected on the TLC plate using autoradiography. Peaks corresponding to the parent tracer and radio-metabolites could be distinguished in the line profile through each sample (*n* = 8) in the autoradiographic image. Significant intersubject and intra-subject variability in radio-metabolites production could be observed with both tracers. For [^18^F]FEPPA, 50% of plasma activity was from radio-metabolites as early as 5-min post injection, while for [^18^F]FAZA, significant metabolites did not appear until 50-min post. Simulation study investigating the effect of radio-metabolite in the estimation of kinetic parameters indicated that 32–400% parameter error can result without radio-metabolites correction.

**Conclusion:**

TLC coupled with autoradiography is a good alternative to high-performance liquid chromatography for radio-metabolite correction. The advantages of requiring only small blood samples (~ 100 μL) and of analyzing multiple samples simultaneously, make the method suitable for individual dynamic PET studies.

## Background

To derive molecular/metabolic information from dynamic positron emission tomography (PET), a kinetic analysis of the radiolabeled tracer is required. Obtaining the time concentration curve of the radiotracer in blood plasma, the arterial input function (AIF), is crucial to accurately portray the pathophysiology. One frequently used method is to sample arterial blood serially and use a radiation detector to measure the activity in the blood samples. The detector only detects the annihilation photons from the decaying positron-emitting isotope and cannot distinguish whether the radionuclide remains attached to the parent tracer or its metabolites [1]. AIF can also be image derived obtained by measuring the activity in an arterial region in dynamic PET. Regardless of the method, measuring the activity in blood could overestimate the AIF because of the metabolite activity. Without correcting for the metabolite activity, results from kinetic analysis based on the overestimated AIF would be erroneous.

The metabolites can be separated from the parent tracer using chromatographic technique like thin layer chromatography (TLC), high-performance liquid chromatography (HPLC) [2, 3] or micellar chromatography [4]. Before analysis by liquid chromatography techniques, the samples would require pretreatment like solid-phase extraction or protein precipitation to prevent clogging of the columns or to prevent protein binding [1, 4]. These preanalysis preparations require a high amount of manual manipulation, which may pose a safety hazard from routine use [5]. A faster method that does not require sample preparation and hence less radio-sample exposure is column switching HPLC. However, the build-up of pressure in the column limits the number of samples to be injected into the column [6]. HPLC is widely used in analytical chemistry and pharmaceutical industry and research to determine the purity of samples. It has high resolution between metabolites and parent tracer with high sensitivity in radioactivity detection due to the use of a scintillation detector coupled with a photo-multiplier tube [7]. However, as a serial analyzer, HPLC can only analyze one sample at a time which can take up to 20 min for each sample. These instruments rely on finely tuned pumps, sensitive detectors and various separation media. This results in high initial purchasing and upkeep costs. Finally, HPLC separation is susceptible to impurities in the solvent mobile phases [1].

An economical alternative to HPLC is TLC which is a simpler version of HPLC. It is not susceptible to impurities, and multiple samples can be analyzed at the same time. The major drawbacks are that TLC suffers from poorer analyte resolution and requires a very sensitive detector to detect analytes at low concentration on the TLC plate. Different techniques have been developed in the past for radio-TLC. Earlier techniques include the zonal analysis [8] and autoradiography technique where the TLC plate is directly exposed to X-ray film [9]. Later, radio-scanners were developed that measured radioactivity at 1–2 mm steps. These techniques have poor analyte resolution (albeit from the intrinsic TLC characteristics), low sensitivity (MBq/mL range), and usually require long exposure time from hours to months for low activity samples and are prone to error [7, 9]. Therefore, our objective was to explore a different detection system with improved sensitivity and time efficiency for radio-TLC. Furthermore, the effect of radio-metabolites in the blood plasma on the accuracy of parameters estimated by kinetic analysis of dynamic PET was assessed using simulation study.

## Methods

### Beaver autoradiography system

**Fig. 1:**
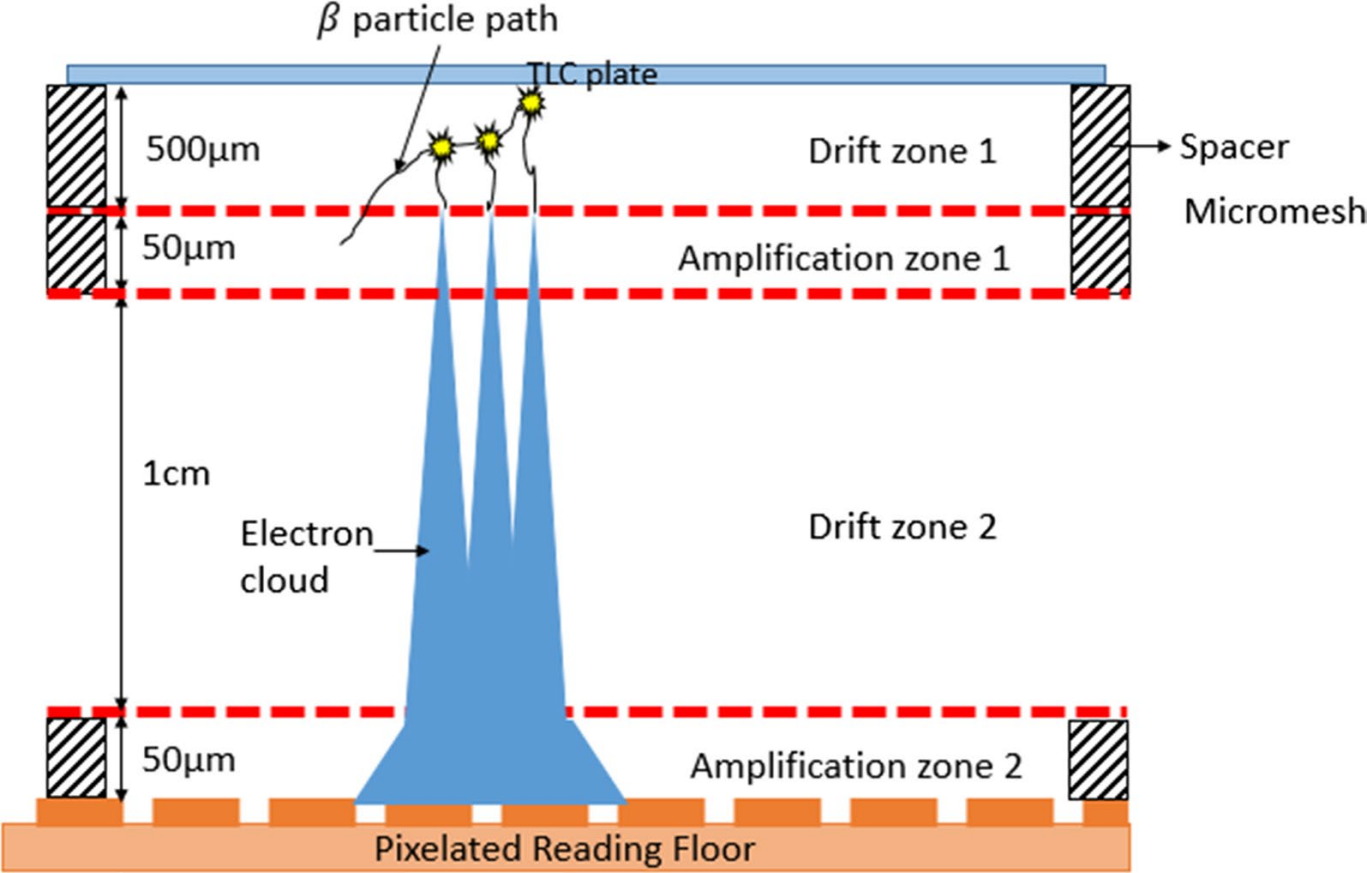
Schematic of beaver autoradiography. Working principle of Beaver MPGD for β-particles. (Adapted from J Instrum. 2009;4:1–9 [11])

Beaver autoradiography (ai4r, France) is a multimodality real-time digital autoradiography system that can image beta and alpha particles [10]. The detector is based on the principle of micropattern gaseous detector (MPGD) using 90% Ne and 10% CO_2_ gas supply which is commercially available in compact gas cylinders. The one we used was designed for imaging large samples with high spatial resolution of 50 µm and sensitivity of 5 × 10^–4^ cpm mm^−2^ [11]. It requires a continuous supply of the gas mixture at a pressure of 1.1 bar. The detector is comprised of two drift zones alternating with two amplification zones separated by nickel micromeshes (Fig. 1). The special feature of the drift zone is the low electric field (1 kV/cm) that guides the electrons from the site of ionization by beta particles from radionuclide decay into the amplification zone [12]. Electrons are multiplied by avalanche effect in the amplification zone due to kinetic energy imparted by the high electric field. The amplification zones are shallow in depth (50 μm) to limit the spread of electron avalanche (cloud) and hence improve spatial resolution [13]. The TLC plate is used as the cathode of the detector and serves as the back end of the first drift zone to prevent back flux of electrons. The electron cloud exiting the second amplification zone is captured by the pixelated reading anode.

### Animal protocol

All experimental procedures were approved by and performed in accordance with guidelines of the Animal Ethics Committee of the University of Western Ontario. Five farm pigs were procured from a farm nearby, and two athymic Rowett Nude (RNU) rats were purchased from Charles River (Saint Constant, Quebec, Canada). The C57BL/10 J mouse used in optimization of [^18^F]FEPPA mobile phase was purchased from Jackson Laboratory (Maine, USA). The animals were under no dietary restriction with free food access before each experiment. Pigs were first anesthetized with Telazol intramuscular injection (1 mL/kg), while rats and mouse by masking with 5% isoflurane, and maintained using isoflurane at 2–3% balanced oxygen and medical air. Pigs (33.7 ± 9.33 kg) were used for [^18^F]FEPPA (21.9 ± 6.34 MBq/kg) analysis. RNU rats (309.75 ± 29.64 g) were used for [^18^F]FAZA (49.54 ± 9.39 MBq/kg) analysis. The tracers were manufactured at the cyclotron/radiochemistry facility of our institution following published procedures [14, 15]. Blood samples were drawn at 8 time points post tracer injection (p.i.)—5, 10, 15, 20, 30, 40, 50 and 60 min. Each rat underwent two blood draws, two weeks apart, to make up a total of 4 sets of rat blood samples. For rats, blood (0.2 mL) was drawn for each sample from a tail vein using heparinized syringe into plasma separator tube. Due to the larger total blood volume of pigs, 2 mL of blood was drawn for each sample from a cephalic vein into EDTA-coated tubes.

### Blood preparation for metabolite analysis

The blood samples were immediately placed on ice to prevent further catabolism. Within 1–2 min after the last sample was taken, all samples were centrifuged at 1,000G in Sero-fuge II centrifuge (Clay-Adams Company, Inc.) for 5 min. The supernatant plasma was aspirated for radio-metabolite analysis.

### Thin layer chromatography (TLC) preparation

Silica-coated TLC plates with F_254_ fluorescent indicator were purchased from MilliporeSigma. Each plate was scored to a height of 9 cm to fit the 13 × 9 cm holder of the Beaver TLC detector. Blood plasma (2 µL) from each blood sample was spotted 1 cm from the bottom of the plate with a micropipette. For optimal use of each imaging session with the TLC detector, two 5-cm-wide plates were used. Five samples including one parent tracer reference (0.11–0.30 MBq in 3–5 mL of isotonic saline) can be spotted on each plate. The plate was air-dried after spotting and then immersed into the mobile phase in a beaker, making sure the solution was less than 5 mm high. The beaker was then covered with parafilm wax paper. The TLC plate developed for approximately 15 min until the solvent (mobile phase) front was roughly 1 cm from the top of the plate. The plate was then removed, air-dried and imaged with the autoradiographic detector for 4 h.

### Optimization of the mobile phase

Using different volume fractions of ethyl acetate, methanol and hexane, the mobile phase was optimized for each tracer. The solution that allowed the least polar analyte to migrate furthest away from the spotting baseline as well as giving a good separation of the metabolites from the unmodified tracer in the autoradiography image was selected as optimized solution. Due to poor analyte resolution with TLC, the plasma metabolites did not appear as discrete spots. [^18^F]FEPPA and [^18^F]FAZA were optimized with blood from a mouse and a pig, drawn at 90 min and 60 min p.i., respectively. In this study, with the optimized mobile phase (solvent), the parent tracer was always closest to the solvent front after the TLC plate was developed.

### Image analysis

Autoradiography images were analyzed with Analyze 12.0 (Analyze Software System). Line profiles were generated by summing the detected counts in a 7-mm segment centered on the sample “track” at 10-mm intervals.

For line profiles where the adjacent metabolite peak overlapped with the parent tracer peak, the area underneath the latter was estimated with a custom-developed program using MATLAB (The MathWorks, Inc.). These line profiles were fitted with two Gaussian functions. The parent tracer peak area was determined as the area of the fitted Gaussian between the limits of μ ± 1.96 σ, where μ is the mean and σ is the standard deviation. For spots where the adjacent metabolite peak did not overlap with the parent tracer peak, the latter was fitted with a Gaussian function and the parent tracer peak area was similarly determined as for the case of overlap.

The fraction of the parent tracer was calculated using the formula:

$${\text{fraction}} = \frac{{{\text{area}}\, {\text{under}}\, {\text{native}}\, {\text{tracer}}}}{{{\text{total}}\, {\text{area}}\, {\text{under}}\, {\text{line}}\, {\text{profile}}}}$$

Each estimated parent tracer fraction for different times p.i. was compared to published literature values for validation.

### Effect of radio-metabolites on kinetic parameter estimation

The kinetic parameters associated with the tracer uptake are obtained by deconvolving the AIF of the parent tracer from the measured tissue concentration curve or tissue time activity curve (TAC). A simulation study was performed to observe the effect of blood plasma radio-metabolites in the estimation of kinetic parameters. For simulating the tissue TAC, our in-house flow-modified two-compartment (F2TC) model [16] that models the bidirectional permeation of the endothelial barrier during the transit time of the tracer through blood vessels, was utilized. The flow-scaled impulse residue function (IRF_F_(t)) for the model is expressed as:

$${\text{IRF}}_{{\text{F}}} \left( t \right) = \left\{ {\begin{array}{*{20}l} F \hfill & {0 \le t < W} \hfill \\ {{\text{Ge}}^{{ - \alpha \left( {t - W} \right)}} + {\text{He}}^{{ - \beta \left( {t - W} \right)}} } \hfill & {t \ge W} \hfill \\ \end{array} } \right.$$

where *F* is the blood flow, *W* is the mean transit time through blood vessels, and *G*, *H*, $\alpha$ and $\beta$ are the fitting parameters obtained by iteratively fitting tissue TAC with nonlinear “interior point” optimization technique. The model’s explicit parameters can be calculated from the fitting parameters as follows:

$$\begin{aligned} K_{1} & = G + H;\quad k_{2} = \frac{G\alpha + H\beta }{{G + H}} \\ k_{3} & = \frac{{GH\left( {\alpha - \beta } \right)^{2} }}{{\left( {G + H} \right)\left( {G\alpha + H\beta } \right)}}; \quad k_{4} = \frac{{\left( {G + H} \right)\alpha \beta }}{{\left( {G\alpha + H\beta } \right)}} = \frac{\alpha \beta }{{k_{2} }} \\ \end{aligned}$$

The explicit parameters are the influx (*K*_1_) and efflux (*k*_2_) rate constant of tracer through the blood tissue barrier, and k_3_ and k_4_ are the binding and disassociation rate constant of the parent tracer to and from the target, respectively.

The measured AIF with metabolite contamination, AIF_*m*_, was simulated using Feng’s model [17, 18]:

$${\text{AIF}}_{{\text{m}}} \left( t \right) = \left[ {A_{1} \left( {t - t_{0} } \right)^{\alpha } - A_{2} - A_{3} } \right]{\text{e}}^{{ - \lambda_{1} \left( {t - t_{0} } \right)}} + A_{2} {\text{e}}^{{ - \lambda_{2} \left( {t - t_{0} } \right)}} + A_{3} {\text{e}}^{{ - \lambda_{3} \left( {t - t_{0} } \right)}}$$

where $$A_{1} = 800, \alpha = 1.0, A_{2} = 20, A_{3} = 20, \lambda_{1} = 4\,\min^{ - 1} , \lambda_{2} = 0.015\,\min^{ - 1} , \lambda_{3} = 0.15\,\min^{ - 1} , t_{0} = 0.15\,\min$$.

AIF_m_ was simulated at 0.5 s and corrected for radio-metabolite by multiplying with the fraction of parent [^18^F]FEPPA measured in “Fraction of parent tracer versus post injection time” section:

$${\text{AIF}}_{{\text{c}}} = {\text{AIF}}_{{\text{m}}} *{\text{fraction}}$$

The corrected AIF_C_ was used to simulated tissue TAC at 0.5 s with ten sets of parameters (Table 1) from patients with high-grade glioma [19]. All the curves were frame averaged according to the following frame schedule: 10 @ 10 s, 5 @ 20 s, 4 @ 40 s, 4 @ 60 s, 11 @ 180 s and 1@ 120 s (total 45 min). The two sets of kinetic parameters estimated by deconvolving AIF_m_ and AIF_c_ from simulated tissue TACs were compared. The difference of the parameters estimated with and without metabolite correction was tested for statistical significance using nonparametric test—either Wilcoxon signed rank or sign test depending on whether the distribution of the differences was symmetric or nonsymmetric, respectively. *P* < 0.05 was declared significant with Bonferroni correction for multiple comparison with 8 parameters ($K_{1} , k_{i} \left( {i = 2,3,4} \right), V_{{\mathrm{p}}} , DV, W, K_{i} )$.

**Table 1:** Ten parameter sets used for simulating the effect of radio-metabolite correction in blood plasma

SET#	${K}_{1}$ (mL min^−1^ g^−1^)	${k}_{2}$ (min^−1^)	${k}_{3}$ (min^−1^)	${k}_{4}$ (min^−1^)	$F$ (mL min^−1^ g^−1^)	*W* (s)	${V}_{\mathrm{p}}$ (mL g^−1^)	DV (mL g^−1^)
1	0.0930	0.5920	0.1840	0.0410	0.37	7	0.043	0.8621
2	0.1370	0.3310	0.2300	0.0700	0.27	7	0.032	1.7738
3	0.0740	0.3440	0.1520	0.0370	0.10	10	0.016	1.0988
4	0.0720	0.4580	0.2880	0.0770	0.29	10	0.048	0.7452
5	0.2220	0.4720	0.1900	0.0870	0.44	5	0.037	1.4975
6	0.1940	0.3280	0.2830	0.1720	0.26	15	0.065	1.5646
7	0.0960	1.0000	0.3060	0.0670	0.38	8	0.051	0.5344
8	0.1010	0.5180	0.3510	0.0750	0.20	10	0.034	1.1075
9	0.4790	1.0000	0.2210	0.1370	0.64	10	0.106	1.2517
10	0.2180	0.4980	0.4480	0.0840	0.87	15	0.218	2.7724

## Results

### Optimization of the mobile phase

**Fig. 2:**
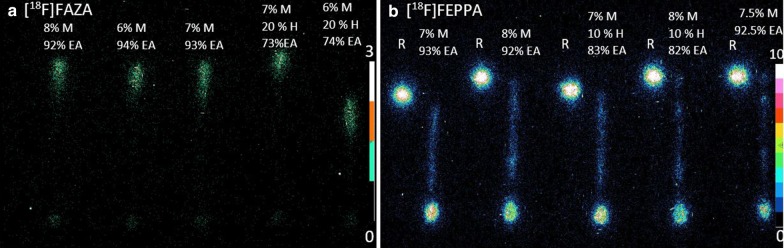
Autoradiographic image of mobile-phase optimization for [^18^F]FAZA and [^18^F]FEPPA. Mobile-phase optimization for **a** [^18^F]FAZA, **b** [^18^F]FEPPA in pig and mouse blood, respectively, at 90 min and 60 min (respectively) post injection, using different fractions of methanol (*M*), and hexane (*H*) in ethyl acetate (EA). With each tracer, five different mixtures were used. For [^18^F]FAZA only plasma samples were used, while for [^18^F]FEPPA each plasma sample was paired with the native tracer in normal saline as the reference (*R*). The optimal mobile phase would spread the radio-metabolites along the entire lane and move the reference furthest from the spotting line. For [^18^F]FEPPA the fourth solution from the left comprised of 8% methanol, 10% hexane and 82% ethyl acetate was optimal, while for [^18^F]FAZA, it was the third solution comprised of 7% methanol and 93% ethyl acetate

The separation of radio-metabolites in blood plasma with different mixtures of methanol, hexane and ethyl acetate for both tracers is shown in Fig. 2. The optimized mobile phase for [^18^F]FEPPA and [^18^F]FAZA was 8% methanol and 10% hexane in ethyl acetate (v/v; fourth solution) and 7% methanol in ethyl acetate (v/v; third solution), respectively.

### Autoradiography

**Fig. 3:**
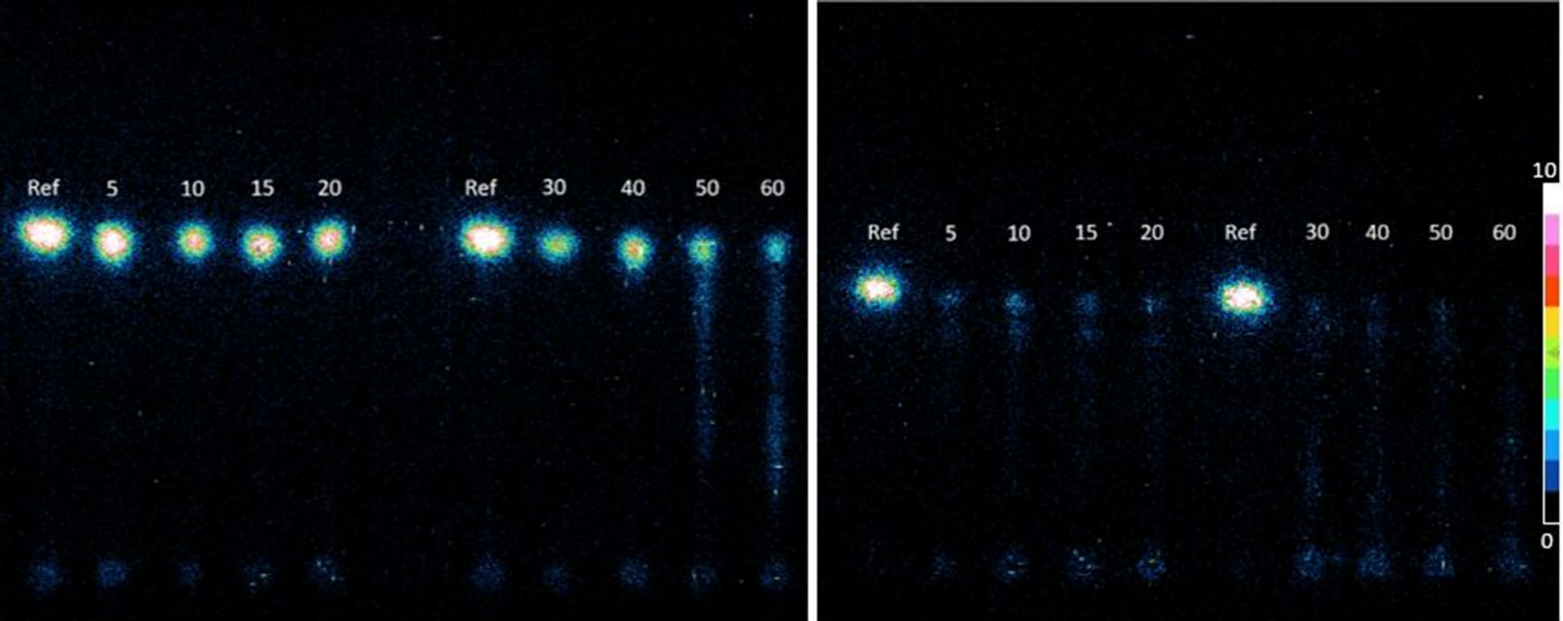
Autoradiographic image of plasma samples. Autoradiography image of plasma samples obtained from a rat injected with [^18^F]FAZA (left) and from a pig injected with [^18^F]FEPPA (right). “Ref” is the reference spot on each TLC plate. The number above each line shows the time in min at which the blood sample was drawn post tracer injection. The direction of motion of the mobile phase (solvent) front from capillary action was from bottom to top. The radio-metabolites that did not move with mobile phase show up as faint “spots” at the bottom along the spotting line

Figure 3 shows the autoradiographic images obtained from TLC plasma metabolite analysis of [^18^F]FAZA (rat) or [^18^F]FEPPA (pig), respectively. Each image showed two TLC plates with the parent tracer in normal saline as reference on each, as well as plasma obtained at different times p.i.

Since the reference parent tracer spot moved the furthest from the spotting baseline, it was the least polar analyte. The spots with similar retention factors (*R*_f_) to the reference spots were the fractions of the parent tracer in plasma at different times p.i. For [^18^F]FAZA on the left, the reference spots’ *R*_f_ was 0.66 ± 0.01. Most of the activity was from the parent tracer, while that at the spotting line could be from the more polar radio-metabolites. Significant conversion of tracer to radio-metabolite was observed from 50 min p.i. For [^18^F]FEPPA on the right, the reference spots’ *R*_f_ was 0.54 ± 0. Radio-metabolites were observed as early as 5 min p.i. as indicated by activity directly below the reference *R*_f_ as well as activity along the spotting line. At 1 h p.i., the parent tracer spot almost disappeared as there was almost complete conversion into metabolites observed as activity all along the track.

### Line profile

**Fig. 4:**
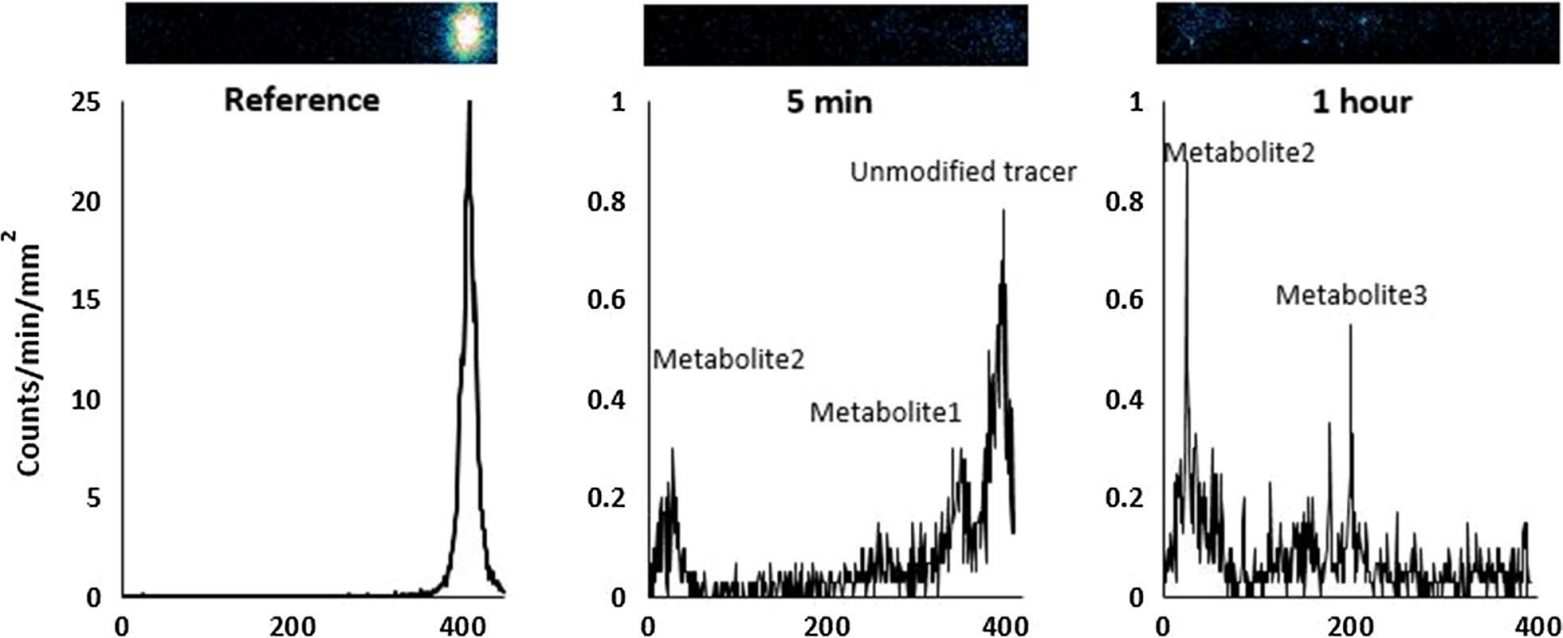
Line profile of [^18^F]FEPPA. Line profile of [^18^F]FEPPA reference, plasma from a pig obtained at 5 min and 1 h post tracer injection. The *y*-axis is detected counts, and the *x*-axis is distance in mm. The corresponding autoradiography image is displayed above the profile. The direction of movement of the solvent front from capillary action was from left to right

Figure 4 shows the line profiles of selected [^18^F]FEPPA spots—reference, 5 min and 1 h p.i.—corresponding to the right image of Fig. 3. For the reference, a well-defined peak was observed due to high signal to noise ratio. At 5 min p.i., three prominent peaks were discernible. The peak on the furthest right was the parent [^18^F]FEPPA, and the peak for the least polar radio-metabolite was close to the parent tracer. The most polar radio-metabolite was located close to the spotting baseline. At 1 h p.i., the parent [^18^F]FEPPA peak was not identifiable. A new peak corresponding to radio-metabolites of intermediate polarity was observed and the amount of the most polar radio-metabolite increased, as indicated by the area. Therefore, the parent [^18^F]FEPPA was almost completely metabolized to radio-metabolites at 1 h p.i.

### Fraction of parent tracer versus post injection time

**Fig. 5:**
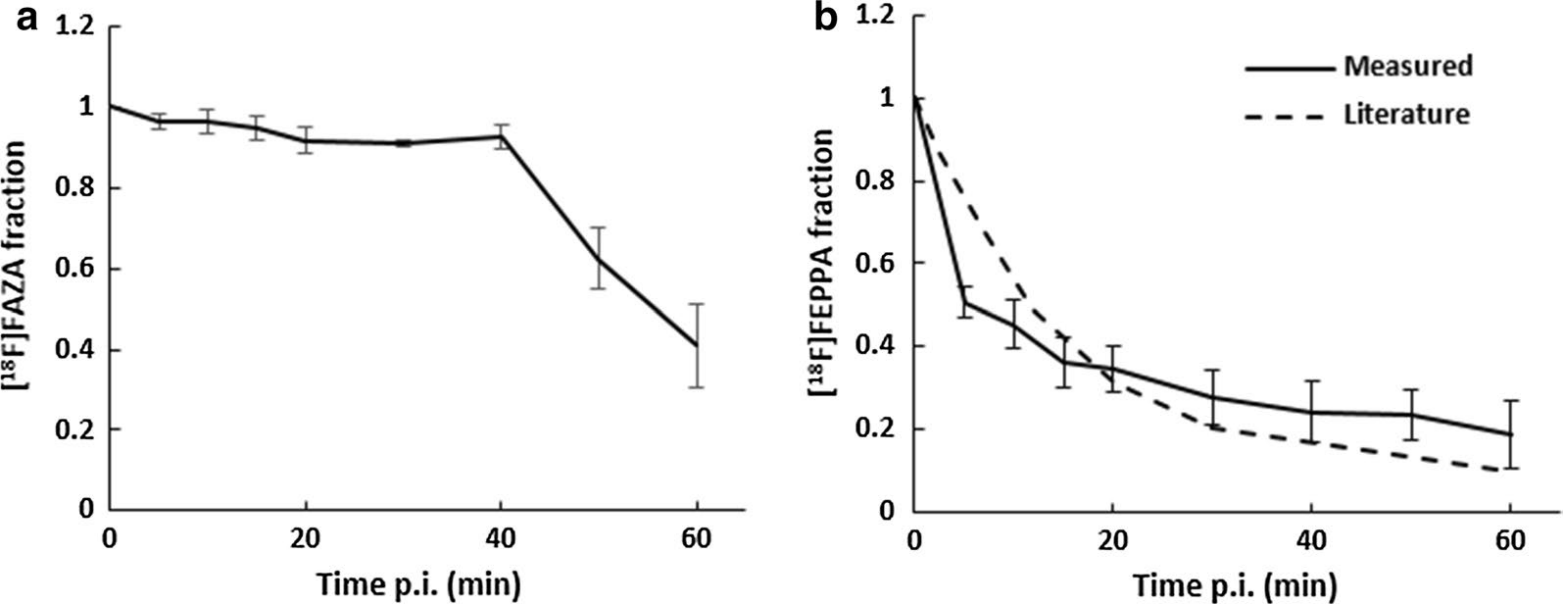
Fraction of parent tracer for [^18^F]FAZA and [^18^F]FEPPA. Native tracer fraction vs time post injection (p.i.) for **a** [^18^F]FAZA, **b** [^18^F]FEPPA. The dashed line in **b** was native tracer fraction from [18]. The error bar corresponds to standard deviation for 5 pig blood samples ([^18^F]FEPPA) and 4 rat blood samples ([^18^F]FAZA)

Figure 5 shows the fraction of parent [^18^F]FAZA and [^18^F]FEPPA in blood at different times post injection. For [^18^F]FAZA, the fraction of parent tracer remained relatively constant at 91% until 40 min p.i. The fraction then decreased to 62% and 40% at 50 and 60 min p.i., respectively. On the other hand, close to 50% of activity in blood was from [^18^F]FEPPA metabolites as early as 5 min p.i. and the parent tracer fraction decreased to 19% at 1 h p.i. Table 2 shows the coefficient of variation (CoV) of the parent tracer fraction arising from intersubject variability. For [^18^F]FEPPA, CoV ranged from 0.07 to 0.43 while [^18^F]FAZA from 0.01 to 0.25.

**Table 2:** Coefficient of variation of parent tracer fraction for [^18^F]FEPPA and [^18^F]FAZA

	5 min	10 min	15 min	20 min	30 min	40 min	50 min	60 min
[^18^F]FEPPA	0.07	0.13	0.17	0.17	0.24	0.32	0.26	0.43
[^18^F]FAZA	0.02	0.03	0.03	0.04	0.01	0.03	0.12	0.25

### Simulation study

**Fig. 6:**
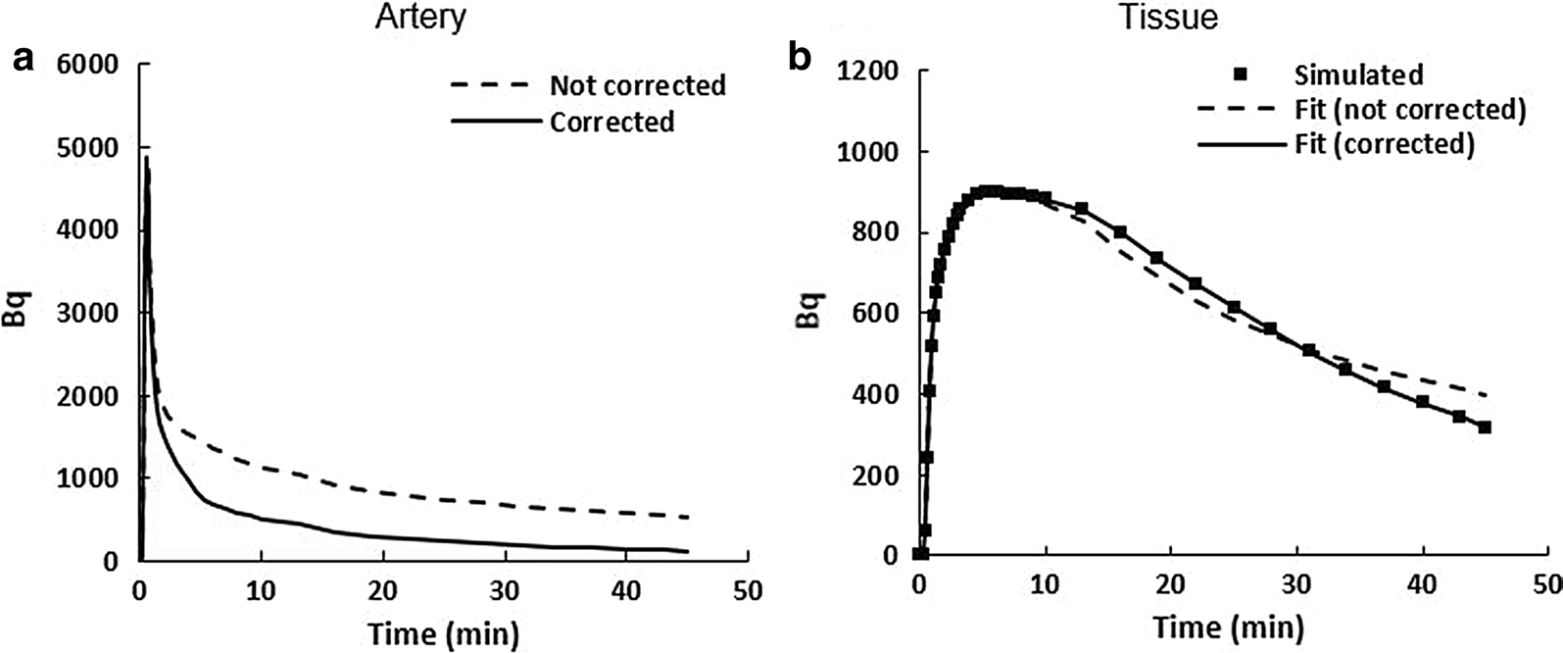
Simulation curves for estimating accuracy of kinetic parameter due to radio-metabolite correction. Curves utilized for simulating the effect of radio-metabolite on kinetic parameter estimation in dynamic PET cases. **a** Arterial curve simulated with Feng’s model for measured (not corrected, dashed line) and radio-metabolite corrected curve (solid line). **b** Tissue curve simulated with parameter set #6 (Table 1, solid square) and fitted curves obtained with measured arterial curve (dashed line) and with radio-metabolite corrected arterial curve (solid line)

Due to conversion of parent tracer to radio-metabolite, metabolite correction lowered the AIF as p.i. time increased (Fig. 6a). When AIF_C_ was deconvolved from the tissue TACs, the parameters estimated were statistically different from those estimated by deconvolving AIF_m_; errors greater than 32% were observed for all parameters (Table 3).

**Table 3:** Median differences in parameters estimated from simulation study

Parameter	*K*_1_ (mL min^−1^ g^−1^)	*k*_2_ (min^−1^)	*k*_3_ (min^−1^)	*k*_4_ (min^−1^)	*V*_p_ (ml g^−1^)	DV (ml g^−1^)	*W* (min)
Median difference	0.003	− 0.124	0.111	− 0.052	0.004	0.806	0.021
*P*	0.75	0.004	0.005	0.004	0.013	0.004	0.083
Bias (%)^a^	198.3	− 32.7	115.4	− 390.3	109.2	81.2	181.0

Median differences between parameters in Table 1 estimated using AIF with and without metabolite correction. *P* value is estimated by nonparametric paired test

^a^Although the differences in the estimated parameters were not normally distributed, the percentage bias was used to approximate the expected error

## Discussion

In this study, we established an alternate method to HPLC to determine the fraction of radio-labeled parent tracer at different times p.i. based on inexpensive TLC and a sensitive beta detector. Fraction of unmetabolized [^18^F]FAZA and [^18^F]FEPPA in normal healthy rats and pigs (respectively) p.i. was measured and compared to literature values, if available. There were large variations in the rate of metabolite production with the same tracer (either [^18^F]FAZA or [^18^F]FEPPA) and between the two tracers in the same and different animals. CoV of parent tracer fraction in blood could be as high as 43%. In addition, simulation study investigating the effect of radio-metabolite correction in measured arterial curve suggested that large error (30–400%) can result in the estimation of kinetic parameters if correction was not incorporated.

The acquired autoradiography images showed clear distinction between radio-metabolites and the parent tracer. The large signal difference between reference and plasma sample was due to 6.7 times difference in the activity between the two. Reference [^18^F]FEPPA (parent tracer in normal saline) was spotted with activity of 126 ± 17 Bq in 2 uL, while plasma samples from pigs were lower in activity—at 5 min p.i. the activity was approximately 17 Bq in 2 uL. For our metabolite studies, either 15.2 ± 1.8 MBq (41–59 MBq/kg) or 427–1216 MBq (13–27 MBq/kg) was administered for the rat ([^18^F]FAZA) and pig ([^18^F]FEPPA), respectively, at the time of injection. These were lower than other published metabolite studies in mice where doses ranging from 20 to 30 MBq (1 GBq/kg) [20] to as high as 68 MBq (3.4 GBq/kg) [21] of tracer were administered due to the lower sensitivity of the radiation detector used. In our studies, even with > 77 times less dose (normalized to body weight to account for the body mass of different species), peaks corresponding to the parent tracer could be distinguished from radio-metabolites. In the few cases where radio-metabolites overlapped with the parent tracer because of similar polarity and hence strength of adhesion to the silica media, the parent tracer peak could be adequately resolved by the curve fitting procedure discussed in “Image analysis” section. Taken the above results together, our method of combining TLC and the Beaver proprietary beta particle detector has the analyte resolution and sensitivity for blood metabolite determination for both [^18^F]FAZA and [^18^F]FEPPA in individual large (pig) or small (rodents) animals. Nevertheless, by comparing the [^18^F]FEPPA dose used in our pig studies (13–27 MBq/kg) to the published patient dose (2.5–6.2 MBq/kg [22–25]) the sensitivity of the detector has to be increased by at least 5 times for the method to be used for blood metabolite determination in individual patients.

From the [^18^F]FEPPA results (Table 2), intersubject variation was observed with more pronounced variability at later time points p.i in agreement with other studies [22, 26, 27] showing intersubject variabilities. This supports our view that the current practice of using a population average in normal subjects to correct for metabolite contamination [26] is not optimal for kinetic analysis and there is a need to determine blood metabolite in individual studies. Furthermore, simulation study (as shown in “Appendix”) validated the requirement of individualized metabolite correction. With our method, multiple samples can be analyzed together within one imaging session, and the exact number of samples depends on the detector size. Currently, we can analyze 8 samples, but with a larger detector size, the number of samples can be increased to 12 or more making it feasible for individualized radio-metabolite analysis.

The effect of not correctly accounting for blood radio-metabolite was investigated with computer simulation using a previously published kinetics model [16]. For all parameter sets listed in Table 1, the fitting to the simulated tissue time activity curve (TAC) failed when radio-metabolite contamination was not corrected for in the arterial TAC. This failure led to large errors (30–400%) in parameter estimation and possible misinterpretation of the tracer pharmacokinetics. For instance, distribution volume of [^18^F]FAZA is related to the amount and activity of nitroreductase present in hypoxic tissue [28], while that of [^18^F]FEPPA is related to density and activity of translocator protein (TSPO) found on the outer mitochondrial membrane [29], particularly within activated immune cells [30]. Additional simulations were performed to investigate how intersubject variability in parent tracer fractions over time would affect the accuracy of the estimated kinetic parameters. As indicated in Additional file 1: Fig. S1, the percentage bias of the same parameters as in Table 3—${K}_{1}, {k}_{2}, {k}_{3}, {k}_{4}, {V}_{{\mathrm{p}}}, DV,$ and $W$, differs widely without and with correction for the different parent tracer fractions. Therefore, individualization of metabolite correction is necessary for accurate measurement of kinetic parameters in dynamic PET. In this study, the F2TC model [16] was used since it incorporates the bidirectional permeability of the blood-tissue barrier during the vascular transit time. The failure to properly model the transit time could introduce additional biases in parameter estimation.

Our measured fractions of parent [^18^F]FEPPA in blood over time p.i. agreed well with those obtained by Rusjan et al. [22]. On the other hand, measured fractions of parent [^18^F]FAZA over time p.i. were not found in literature. Studies of [^18^F]FAZA by Verwer et al. showed that only 10% of the activity in blood was from metabolites at 70 min p.i. [24] with the use of solid-phase extraction and HPLC. Our study showed that significant metabolite fraction (~ 10%) in blood started at 40 min and increased to 60% at 60 min p.i. Jans et al. [20] also used TLC to estimate the metabolite fraction in blood, and no metabolite was observed. However, there were two mitigating factors with their experiments that could explain the difference in the measured metabolite fraction in blood. First, it was not known whether the mobile phase used was optimized for the tracer; second, the detector used may not be as sensitive as our one (Additional file 1: Fig. S1).

The time required for blood metabolite analysis using our method starting with the collected blood samples (excluding the image processing time) comprised of: 5 min of centrifugation, 5 min for spotting samples on and drying the TLC plate, and 15 min of TLC plate development for a total of 25–30 min. Technically, the solvent front is required to move beyond the furthest point the samples or the parent tracer moved during development. In our experiments, the parent tracer (either [^18^F]FAZA or [^18^F]FEPPA) which moved the furthest, moved approximately 4.5 cm, while the solvent front moved over 7 cm. Therefore, the development time can be shortened to 10 min. The autoradiograph image was acquired over 4 h in this study. However, 1-h acquisition was tested, and the acquired image showed good image quality (signal-to-noise ratio) as shown in Additional file 2: Fig. S2. Similar to HPLC with automated injector, once the blood samples are prepared, all the samples can be analyzed/imaged within an hour without an operator.

Other comparative similarities and differences between our method and HPLC are listed in Table 4.

**Table 4:** Differences and similarities between radio-HPLC and TLC-autoradiography

Properties	Radio-HPLC	TLC—autoradiography
Volume of plasma required	1–2000 $\upmu \mathrm{L}$ [31]	2 $\upmu \mathrm{L}$
Mobile-phase optimization	Required	Required
Analyte resolution	High	Low—overlap of analyte peaks can be resolved by Gaussian fitting
Analyte identification^a^	Yes	No
Sample analysis mode	Serial	Multiplex
Operation	May require separate HPLC column for each tracer	Single use inexpensive TLC plate for each run
	HPLC column requires regular washing to prevent clogging and removal residual analytes from previous runs [1, 32]	Single use TLC—no maintenance is required
Tissue section autoradiography	No	Yes

^a^The chemical identity of the radio-metabolites is not required for metabolite fraction correction

Taking all the above comparative advantages and disadvantages of our method and HPLC into consideration, we conclude that our method could be more suited for individualized metabolite measurement in blood than HPLC. Note that independent of whether the AIF is measured with timed arterial blood sample or is image derived by measuring the activity in an arterial region in dynamic PET images, metabolite correction is required.

As shown in autoradiography images, the resolution is poor which could be improved by altering the pH of the mobile phase. Since the silica stationary phase tends to be acidic, adding trialkyl amines can improve the resolution of basic moieties present in the analytes. Similarly, addition of a small percentage of acetic or formic acid can reduce so-called streaking of acidic moieties. Additionally, alternative stationary phases such as alumina or cellulose could be used to improve resolution of individual metabolites. Ultimately, as percentage of parent fraction is the main outcome measurement from TLC analysis, these optimizations may prove unproductive [33]. Furthermore, the signal to background ratio of the autoradiographic image can be improved by injecting higher amount of radioactive tracer.

There are several limitations with our study. The measured blood metabolite fraction was not validated against the reference HPLC method. However, our [^18^F]FEPPA results agreed with literature values measured with reverse phase HPLC (Fig. 6b). The number of blood samples used for each tracer was small. Even with this small number of animals, the intersubject variability in metabolite fraction was prominent (Table 2), suggesting that this result could be the true in vivo situation and the importance to measure metabolite fraction for each individual subject. We investigated only two tracers, [^18^F]FEPPA and [^18^F]FAZA, as examples. Since analyte separation of TLC depends on the polarity of the tracer and its metabolites, for other tracers the mobile phase will have to be optimized. We have tested our method only with the ^18^F radionuclide. Since other common PET radionuclides including ^11^C, ^13^ N, ^68^ Ga and ^89^Zr emit β^−^ particles in their decay, our method would also work except, like the mobile phase, the limit of radioactivity detection must be determined for each radionuclide separately.

## Conclusion

We were able to measure the fraction of parent radiolabeled tracer in blood after it was injected into the body using TLC and the Beaver autoradiography system. This fraction is required to correct the AIF obtained by measuring the activity in timed arterial blood samples or in arterial region in dynamic PET images. Without this correction, the AIF will be overestimated leading to errors in the kinetic analysis of dynamic PET. Although we used two specific tracers, [^18^F]FAZA and [^18^F]FEPPA, to develop the method,the system can be used for other tracers by optimizing the mobile phase for each of them. Due to its capability to analyze multiple (> 8) blood samples at the same time with preparation time as short as 25–30 min, our method will enable individualize blood metabolite correction for kinetic analysis of dynamic PET.

## Supplementary information

**Additional file 1.** **Fig. S1.** Median differences in kinetic parameters estimated without and with metabolite correction according to the parent tracer fractions shown in Figure S1. Parameter numbers 1-7 correspond to the list of kinetic parameters - $K_{1} , k_{2} , k_{3} , k_{4} , V_{{\mathrm{p}}} , DV,$ and $W$.

**Additional file 2.** **Fig. S2.** Comparison of autoradiography image of 1-hr vs 4-hr acquisition duration. Image of blood plasma obtained from a rat injected with [^18^F]FAZA. The SNR of image (a) acquire for 1 hour is acceptable with discernible spots for native tracer and radio-metabolites. (b) The same TLC image that is acquired for four hours immediately after (a) was acquired. The bright spot is the reference “ref” parent tracer in saline followed by blood samples drawn at 30, 40, 50 and 60 minute post injection.

**Additional file 3.** **Fig. S3.** Different parent tracer fractions over time to simulate inter-subject variability. Different parent tracer fraction over time curves were simulated from that measured for [^18^F]FEPPA (Figure 5(b)) by contracting and expanding the time axis by different scale factors.

## Data Availability

The datasets used and/or analyzed during this study are available from the corresponding author on reasonable request.

## Appendix

### Simulation study to evaluate the effect of different time metabolite activity in the blood plasma on kinetic parameter estimation

To demonstrate how different metabolite activities in blood plasma affects the accuracy of estimated kinetic parameters, different time parent tracer fraction curves arising from intersubject variability were simulated from the measured [^18^F]FEPPA fraction curve in Fig. 5b and simulation studies were repeated according to “Effect of radio-metabolites on kinetic parameter estimation” section.

### Methods

Different time parent tracer fraction representing intersubject variability was simulated by expansion and contraction of the time (*x*-axis) axis of the measured [^18^F]FEPPA fraction, as determined from “Fraction of parent tracer versus post injection time” section. The function describing the fraction of parent [^18^F]-FEPPA over time was estimated by fitting the fraction with a biexponential curve as shown below:

$$\mathrm{fraction}\, (t)=A{e}^{-\alpha t}+B{e}^{-\beta t}$$

According to the fit, the fraction was best described with *A* = 0.5769, *B* = 0.4442. $\alpha =0.2657$, $\beta =0.0140$. The expansion and contraction of the curve was accomplished by multiplying the time variable with a scale factor, *k*:

$$\begin{aligned} & t = kt^{\prime} \\ & {\text{fraction}}^{\prime}\,\left( t \right) = {\text{Ae}}^{{ - \frac{{\alpha t^{\prime}}}{k}}} + {\text{Be}}^{{ - \frac{{\beta t^{\prime}}}{k}}} \\ \end{aligned}$$

where $k$ can either be < 1 or > 1 to represent contraction and expansion, respectively, and ${\text{fraction}}^{\prime}(t)$ is the modified parent tracer fraction. For each simulated ${\text{fraction}}^{\prime}(t)$, the simulation was performed in a similar fashion to “Effect of radio-metabolites on kinetic parameter estimation” section.

### Results

Additional file 3: Fig. S3 shows the [^18^F]FEPPA fraction with varying $k$ values. $k = 1$ corresponds to the measured [^18^F]FEPPA fraction shown in Fig. 5b, and it is also the average of the parent fraction of $k=0.8, 0.9, 1.1$ and $1.2$, indicating that it simulates the population average. As indicated in Fig. 2, the percentage bias of the same parameters as in Table 3—${K}_{1}, {k}_{2}, {k}_{3}, {k}_{4}, {V}_{{\mathrm{p}}}, DV,$ and $W$, as estimated by AIF_m_ and AIF_c_, differs widely among the different scale factors, *k*. Therefore, as advocated in Discussion, individualization of metabolite correction is necessary for accurate measurement of kinetic parameters in dynamic PET.

## Abbreviations

AIF: Arterial input function
CoV: Coefficient of variation
DV: Distribution volume
*F*: Blood flow
F2TC: Flow-modified two compartment
HPLC: High-performance liquid chromatography
IRF: Impulse residue function
*K*_1_: Influx rate constant
*k*_2_: Efflux rate constant
*k*_3_: Binding rate constant
*k*_4_: Disassociation rate constant
*K*_*i*_: Net influx rate
MPGD: Micropattern gaseous detector
p.i.: Post injection
PET: Positron emission tomography
*R*_f_: Retention factor
SPE: Solid-phase extraction
TAC: Time activity curve
TLC: Thin layer chromatography
*V*_p_: Blood volume
*W*: Mean transit time
