# Supplementary figures and images for: Plasma radio-metabolite analysis of PET tracers for dynamic PET imaging: TLC and autoradiography
Source: EJNMMI Res. 2020 Nov 23;10:141. doi: 10.1186/s13550-020-00705-2 (PMC7683627; doi:10.1186/s13550-020-00705-2)

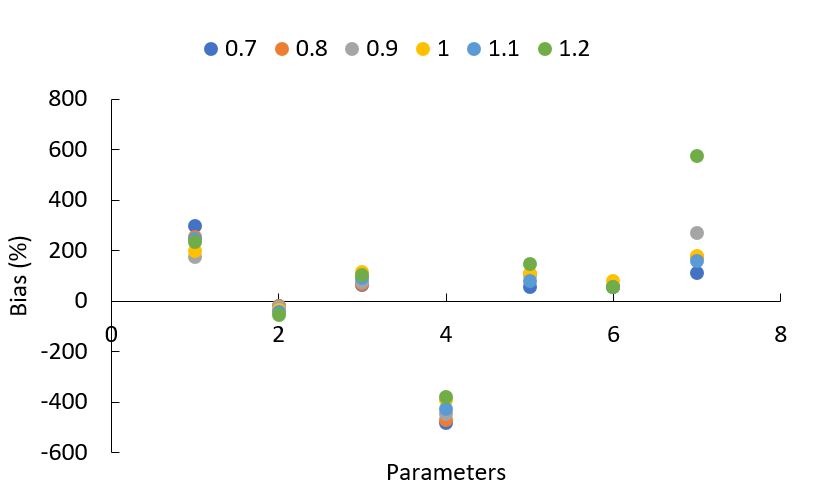

Supplement: Supplementary file 1 — Additional file 1. Fig. S1. Median differences in kinetic parameters estimated without and with metabolite correction according to the parent tracer fractions shown in Figure S1. Parameter numbers 1-7 correspond to the list of kinetic parameters - $K_{1} , k_{2} , k_{3} , k_{4} , V_{{\mathrm{p}}} , DV,$K1,k2,k3,k4,Vp,DV, and $W$W. [file 13550_2020_705_MOESM1_ESM.jpg]

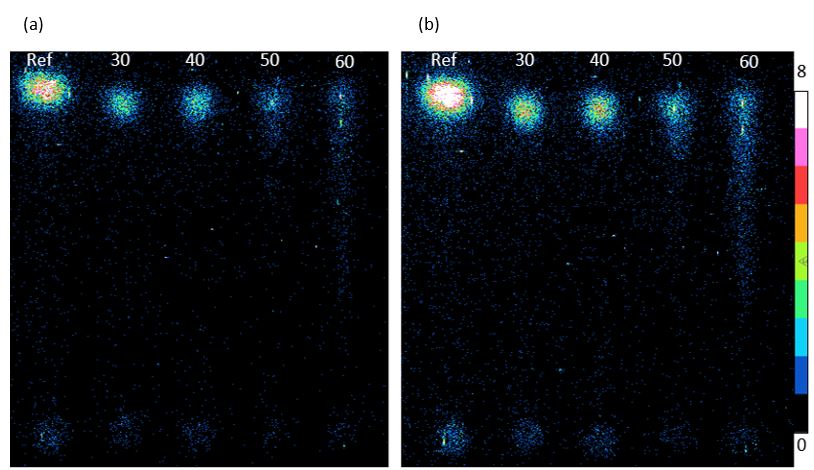

Supplement: Supplementary file 2 — Additional file 2. Fig. S2. Comparison of autoradiography image of 1-hr vs 4-hr acquisition duration. Image of blood plasma obtained from a rat injected with [18F]FAZA. The SNR of image (a) acquire for 1 hour is acceptable with discernible spots for native tracer and radio-metabolites. (b) The same TLC image that is acquired for four hours immediately after (a) was acquired. The bright spot is the reference “ref” parent tracer in saline followed by blood samples drawn at 30, 40, 50 and 60 minute post injection. [file 13550_2020_705_MOESM2_ESM.tiff]

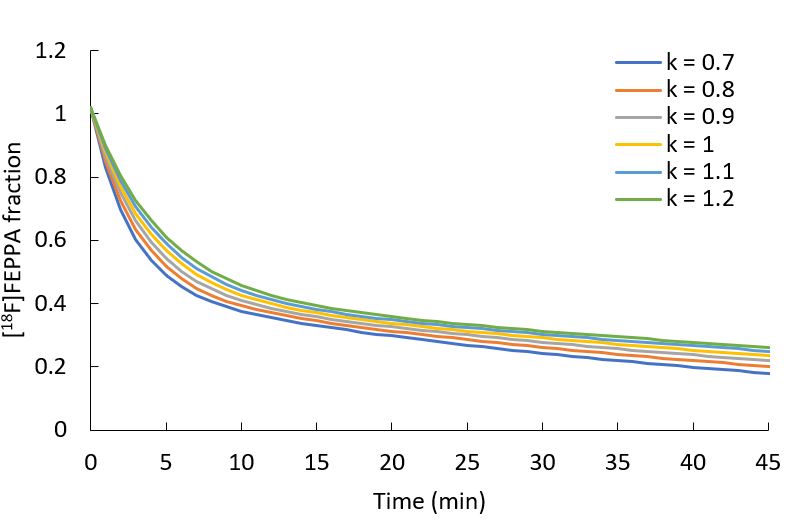

Supplement: Supplementary file 3 — Additional file 3. Fig. S3. Different parent tracer fractions over time to simulate inter-subject variability. Different parent tracer fraction over time curves were simulated from that measured for [18F]FEPPA (Figure 5(b)) by contracting and expanding the time axis by different scale factors. [file 13550_2020_705_MOESM3_ESM.jpg]
